# The penetration efficiency of a dissolved model drug into hair follicles depends on the concentration of added nanoparticles

**DOI:** 10.1007/s13346-024-01718-3

**Published:** 2024-10-04

**Authors:** Loris Busch, Darya Asadzadeh, Anna Lena Klein, Phuvamin Suriyaamporn, Mont Kumpugdee Vollrath, Cornelia M. Keck, Martina C. Meinke

**Affiliations:** 1 Center of Experimental and Applied Cutaneous Physiology, Department of Dermatology, Venereology and Allergology, Charité—Universitätsmedizin Berlin, Corporate Member of Freie Universität Berlin and Humboldt-Universität zu Berlin, Charitéplatz 1, 10117 Berlin, Germany; 2 Department of Pharmaceutics and Biopharmaceutics, Philipps University Marburg, Robert- Koch-Str. 4, 35037 Marburg, Germany; 3 Laboratory Pharmaceutical Technology, Faculty II-Mathematics-Physics-Chemistry, Berliner Hochschule für Technik, Luxemburger Str. 10, 13353 Berlin, Germany; 4 Pharmaceutical Development of Green Innovations Group (PDGIG), Department of Industrial Pharmacy, Faculty of Pharmacy, Silpakorn University, Nakhon Pathom, 73000 Thailand

**Keywords:** Confocal laser scanning microscopy, Ratchet effect, Adhesion effect, Skin penetration

## Abstract

Hair follicles have recently emerged as promising drug delivery targets and gates for skin penetration. The so-called ratchet effect, which is based on an interaction between the hair shaft surface, the intrafollicular stratum corneum and nanoparticles, has proven to be very effective for the transport of active ingredients. Especially the nanoparticle-assisted decolonization of hair follicles constitutes an interesting new area of application. In a recently published work it was shown that small molecules as well as macromolecules solved in an outer phase of a formulation can be transported into the deeper parts of the hair follicles by adding nanoparticles to the formulation. In this case the nanoparticles constitute an entity independent of the drug and the transport is hypothesized to be based on an adhesion effect. In the present work, we focused on the impact of the particle concentration in the formulation on the transport efficiency of the model drug fluorescein sodium into hair follicles utilizing an ex vivo porcine skin model. It was observed that a particle concentration of 4% significantly enhances the transport efficiency of fluorescein as compared to 2% particle concentration. Doubling the concentration to 8% did not significantly increase the penetration depth. The effect evolved more efficiently when using 4 Hz circular motion massage as compared to 100 Hz oscillating massage. These results deliver interesting information on the optimal formulation as well as application parameters for a future application in clinical studies for e.g. skin antisepsis purposes.

**Graphical abstract:**

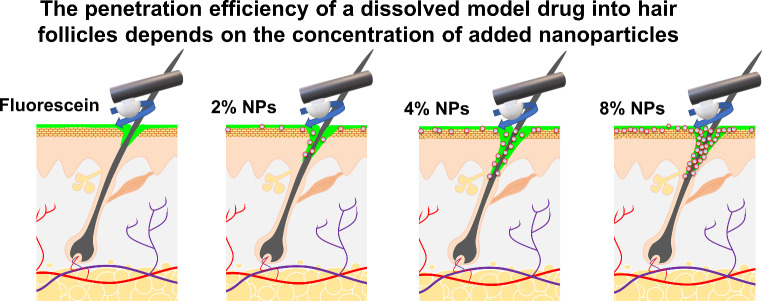

## Introduction

In recent years, hair follicles have been increasingly studied as targets and gates for drug delivery to the skin. Especially the application of nanoparticles (NPs), which are known for their deep penetration into hair follicles constitutes a promising drug delivery approach [1–3]. In addition to the presence of excipients [4], the size of the NPs is one of the main factors for the penetration depth into the follicles, whereby the maximum penetration depth is achieved with NPs of 600 to 700 nm [5]. The penetration of hair follicles by NPs is based on a mechanical effect which is published as the so-called ratchet effect. Here, a massage-induced interaction between the hair shaft surface, the intrafollicular stratum corneum and the NPs in the context of a transverse hair shaft movement is the driving force for NP transport towards the proximal parts of the hair follicle [6]. Reaching the pilosebaceous units with the help of NPs represents a promising approach for therapeutic strategies aimed at optimizing skin antisepsis [7–11] or combating alopecia [12–14]. For the transportation of therapeutic agents, these are usually loaded onto or encapsulated in the corresponding cargos [15–20], which often requires a complex production process with the corresponding costs. Furthermore, it is known that particles would only penetrate the surrounding tissue via the transfollicular pathway from a lower limit of 20–40 nm [1, 21], which would be necessary for many therapeutic approaches. Therefore, a release process is needed, which requires an even higher complexity of the system [22].

Recently Klein et al. presented a novel approach where the transport of dissolved active ingredients into the hair follicles was driven by NPs which were present independently in the formulation [23]. In this way, they were able to show that it is possible to facilitate the transport of active ingredients that are only dissolved in the continuous phase of the formulation, i.e. they are not directly coupled to or incorporated into the particles. The transport mechanism behind this phenomenon is hypothesized to be based on an adhesion effect. For such a drug delivery system the dependency of the particle concentration and the massage frequency is not known.

To determine the optimal particle concentration for reaching deeper areas of hair follicles, in the present study we focused on the effects of particle concentration in the formulation on the transport efficiency of the model drug fluorescein sodium into hair follicles, using an ex vivo porcine skin model.

## Materials and methods

### Preparation of polystyrene nanoparticle formulations

Uncolored polystyrene nanoparticles (PSNPs) (10.1% w/w in deionized water, product number: PS0400CLA-3, VDO Biotech, Suzhou, China) and fluorescent PSNPs (1% w/w in deionized water, product number: 2211709 A, VDO Biotech, Suzhou, China) were used for the experiments. Both types of PSNPs have a mean diameter of 428 nm. Three suspensions of 2% w/w, 4% w/w and 8% w/w PSNP content were prepared using both types of PSNPs (Table 1) in which the fluorescent PSNP concentration was kept constant. Fluorescein sodium (0.2% w/w stock solution, Sigma-Aldrich Chemie GmbH, Steinheim, Germany) was solved in the outer phase of the formulation as a model drug.

**Table 1:** The suspensions consist of uncolored polystyrene nanoparticles (PSNPs) and fluorescent PSNPs. The outer phase of the suspensions was labeled with fluorescent dye fluorescein sodium. Values given as % w/w

	Fluorescein + 8% PSNPs	Fluorescein + 4% PSNPs	Fluorescein + 2% PSNPs	Fluorescein control
Fluorescent PSNPs	0.1%	0.1%	0.1%	-
PSNPs	7.9%	3.9%	1.9%	-
Fluorescein sodium stock solution 0.2%	0.01%	0.01%	0.01%	0.01%
Demineralized water	91.99%	95.99%	97.99%	99.99%

### Size verification of PSNP formulations

Photon correlation spectroscopy (Zetasizer 3000, Malvern Panalytical GmbH, Kassel, Germany) was utilized for NP size determination (temperature: 25 °C, scattering angle: 90°, wavelength: 633 nm). The measurements were carried out in triplicates using 1:1000 dilutions of each suspension. Mean values of the particle size and polydispersity index (PDI) of PSNP suspensions in three different concentrations (8% w/w, 4% w/w, 2% w/w) containing fluorescein sodium are presented in Table 2 for three different time points (1 day, 40 days, 90 days).

**Table 2:** Physical characterization of PSNP formulations in three different concentrations and at three different time points by photon correlation spectroscopy (particle size [nm] and polydispersity index (PDI)). Values given as mean ± standard deviation

	Particle size [nm]			PDI		
Days after production	1	40	90	1	40	90
Fluorescein + 8% PSNPs	481 ± 5	475 ± 2	483 ± 4	0.157 ± 0.034	0.171 ± 0.062	0.181 ± 0.029
Fluorescein + 4% PSNPs	457 ± 4	452 ± 1	441 ± 8	0.177 ± 0.025	0.337 ± 0.090	0.284 ± 0.067
Fluorescein + 2% PSNPs	425 ± 4	429 ± 5	431 ± 5	0.244 ± 0.072	0.278 ± 0.810	0.270 ± 0.031

The deviation of the particle diameter of the prepared suspension with respect to the manufacturer’s specification (428 nm) was 12% (8% PSNP suspension), 6.8% (4% PSNP suspension) and 0.7% (2% PSNP suspension) and the formulations showed a high 3-month stability.

### Preparation of ex vivo porcine ear skin

Ex vivo porcine skin was utilized because of its high similarity to human skin [24–26] and the advantage of the absence of follicular contraction after excision [27]. The preparation of ex vivo porcine ear skin was conducted according to Busch et al. [7]. Porcine ears of six months old donor pigs were obtained from a local butcher. Porcine ears without any visible injuries were selected and the experiments were executed not later than 48 h postmortem. The porcine ears were cleaned under cold tap water, dried with paper towels and stored at 4 °C until the experiments. Pre-experimentally, the porcine ears were flattened and fixed on a polystyrene board by using cannulas. Test areas of 2 cm × 3 cm were labelled using window color (fun & fancy, Marabu GmbH & Co. KG, Tamm, Germany) and the included hairs were shortened carefully.

### Ex vivo application of PSNPs and cryohistological preparation

**Fig. 1:**
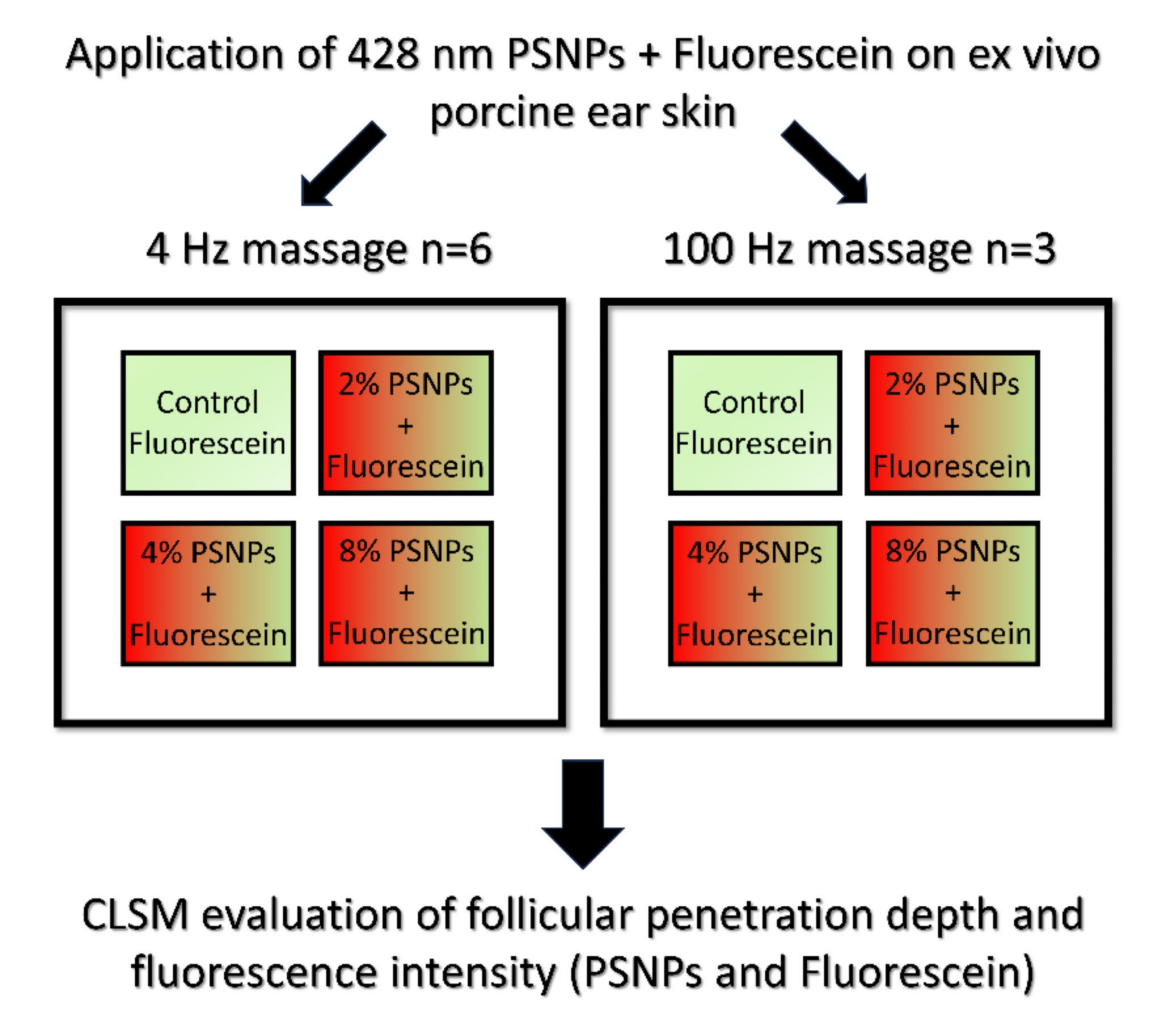
Overview of the experimental design of the study. Polystyrene nanoparticles (PSNPs) were either applied with 4 Hz massage (circular motion) on *n* = 6 ex vivo porcine ears or 100 Hz (oscillating motion) on *n* = 3 ex vivo porcine ears in three different concentrations (2%, 4% or 8% w/w) containing fluorescein as a model drug in the outer phase of all formulations. On a fourth area a particle-free solution only containing fluorescein was applied on each ear. Follicular penetration depth and intrafollicular fluorescence intensity of fluorescein as well as PSNPs were evaluated (*n* = 10 hair follicles per area on each porcine ear) using confocal laser scanning microscopy (CLSM)

Before the formulations were applied to the labeled areas, biopsies of 5 × 5 mm^2^ were taken from an untreated skin area which served as a negative control. A quantity of 20 µl/cm^2^ of each formulation (2%, 4% and 8% PSNPs + fluorescein or fluorescein control) was then applied homogeneously to a total of four marked skin areas. After each application the respective skin area was massaged using a sound wave device (NOVAVON pro, NOVAFON GmbH, Weinstadt, Germany) at a frequency of 4 Hz (*n* = 6 porcine ears) or 100 Hz (*n* = 3 porcine ears) for a period of 2 min (Fig. 1). For 4 Hz, the skin areas were massaged manually in circular motion with a constant pressure massage using the app Pro Metronome (EUMLab Xanin Technology GmbH, Berlin, Germany) while the massage device was switched off. After the massage, the formulation was incubated for 10 min at room temperature. Subsequently, cold spray (Solidofix^®^, Carl Roth GmbH + Co. KG, Karlsruhe, Germany) was sprayed onto the treated skin and 5 × 5 mm^2^ biopsies were taken from each area using a scalpel. The biopsies were transferred to cryotubes and stored at -20 °C. Cryohistological preparation was conducted according to Busch et al. [7].

### Confocal laser scanning microscopy

Confocal laser scanning microscopy (CLSM) (LSM 700, Carl Zeiss AG, Oberkochen, Germany) with a 10x objective was used to examine the sections. Fluorescence images of the formulation of PSNPs and fluorescein were overlaid with brightfield images of the tissue in order to locate the formulation without interference of biological autofluorescence. Negative controls (*n* = 5 untreated hair follicles of each porcine ear) were applied to avoid false positive signal by adjusting the detector gain below the detection limit. The excitation of the fluorophores was carried out with two laser lines. Fluorescein was excited at a wavelength of 488 nm. The maximum emission was detected at 517 nm. The fluorescence of the dyed PSNPs was generated by excitation at a wavelength of 555 nm and detected at a maximum emission wavelength of 593 nm. Image acquisition and measurement of follicular penetration depth of PSNPs as well as fluorescein was conducted via ZEN 2012 software (Carl Zeiss AG, Oberkochen, Germany). For treated areas, *n* = 10 hair follicles were examined. In case of multiple sections per hair follicle, the global maximum of each follicle was considered for data evaluation.

### Image J analysis

Image analysis via ImageJ (Wayne Rasband, National Institutes of Health, Bethesda, MD, USA) was utilized [28] for evaluation of the mean fluorescence brightness of both fluorescence channels (PSNPs and fluorescein) assuming a higher fluorescence brightness correlating with increased concentration.

### Statistical analysis

Statistical analysis was executed via IBM SPSS^®^ Statistics 22 (IBM, Armonk, NY, USA). The Shapiro-Wilk-test was utilized for checking the normal distribution of each group. One-way ANOVA testing with Tuckey post hoc tests was executed for mean value comparison affording a significance of *p* < 0.05. Significances of *p* = 0.05 to *p* = 0.10 were categorized as tendencies and thus written out as full value.

## Results and discussion

**Fig. 2:**
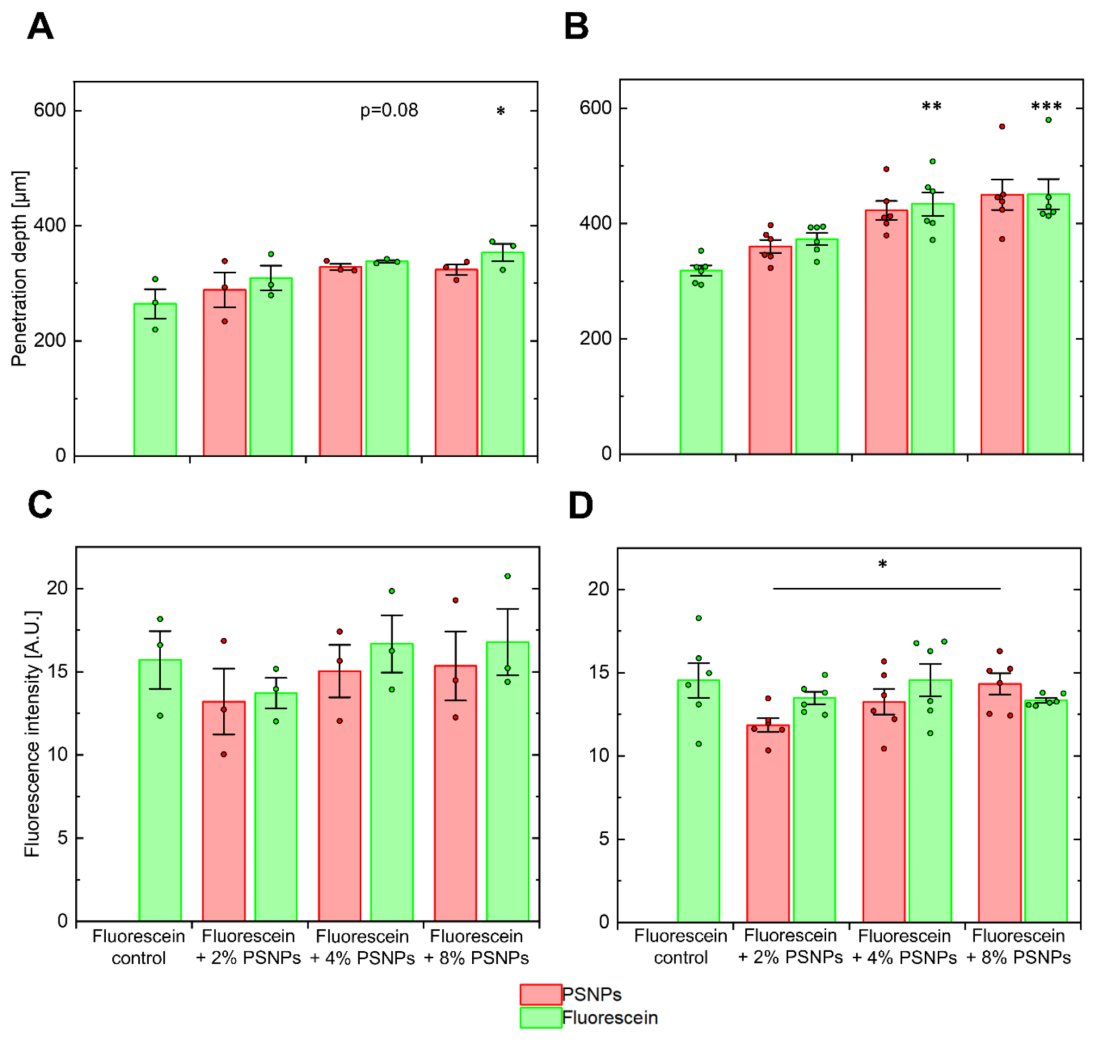
Representation of the follicular penetration depth of fluorescein (green bars) and PSNPs (red bars) after massage at 100 Hz (**A**) and 4 Hz (**B**) as well as the intrafollicular fluorescence intensity after massage at 100 Hz (**C**) and 4 Hz (**D**). A successive increase in the penetration depth of fluorescein compared to a particle-free control is observed with increasing PSNP concentration with a saturation effect occurring between 4% and 8% PSNPs. The total penetration and relative penetration increase is generally higher after massage at 4 Hz (**B**) than after massage at 100 Hz (**A**). These results show that the transport of active ingredients solved in the outer phase into hair follicles is most effective with a PSNP concentration of 4% at a massage frequency of 4 Hz. Despite that, no clear trend was observed with regard to intrafollicular fluorescence intensity, which means that no particle-mediated increase in intrafollicular fluorescein concentration was detectable. *p < 0.05; **p < 0.01; ***p < 0.001

The follicular penetration depth was evaluated after application of fluorescein solutions with additive PSNPs at 4 and 100 Hz. At 100 Hz, there was an increase in the follicular penetration depth of the active substance fluorescein after the addition of 2% PSNPs by 17% compared to the particle-free control, while 4% PSNPs resulted in an increase of 28% (*p* = 0.08, non-significant but statistical tendency present). After the addition of 8%, an increase of 34% was achieved (*p* < 0.05, Fig. 2A). Comparatively low massage frequencies that have no oscillating components are known to produce higher particle penetrations [29]. Therefore, it should also be tested whether this applies to the recently published particle-mediated transport of active ingredients in solution [23]. As expected, the relative and total increase after addition of particles was higher for a massage at 4 Hz for a massage at 100 Hz for particle concentrations > 2%. The reason for this is the lack of a longitudinal component of movement that is present in oscillatory motion, whereas circular motion primarily promotes a transverse movement of the hair shaft in the hair follicle, which is known to be more effective for NP transportation [6, 29]. Furthermore, it is also known that comparably slow movement frequencies of the hair shaft are more effective for the transport of NPs into the hair follicle. Thus, in comparison to the particle-free control, increases of 17% (2% PSNPs), 36% (4% PSNPs, *p* < 0.01) and 42% (8% PSNPs, *p* < 0.001) were reached (Fig. 2B). An increase in the intrafollicular fluorescence intensity representative of the amount of the model drug fluorescein introduced into the hair follicles could not be determined (Fig. 2C and D). Only one significant difference was observed with regard to the fluorescence intensity of the PSNPs (2%<8%, *p* < 0.05, Fig. 2D).

As already presented by Klein et al. [23], the particle-mediated follicle penetration of a dissolved low-molecular compound was also proven in the present study. Numerous studies have already demonstrated the successful transport of active ingredients into hair follicles via NPs [4, 7, 19, 22, 28, 30, 31]. In these studies, though, the NPs were typically loaded with the active or consisted directly of the active ingredient. In the present study, however, the transport of an active ingredient dissolved in the outer phase into the hair follicle, driven by NPs, was demonstrated. As already hypothesized, this is possibly subject to a phenomenon of a dragging effect [23]. Here, the outer phase is dragged into the hair follicle by adhesion to the NPs as part of intrafollicular nanoparticle transport.

**Fig. 3:**
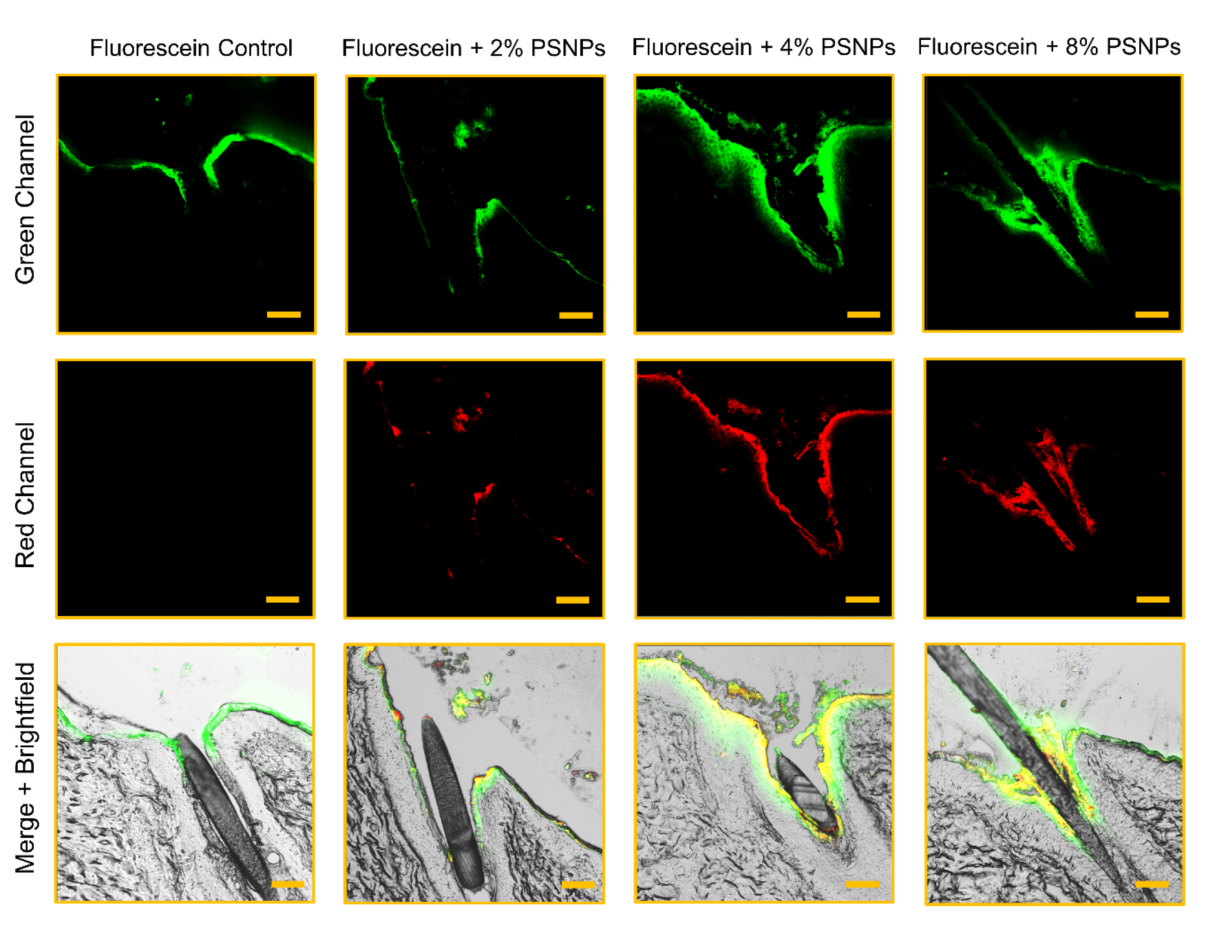
Representative CLSM images in the green channel (first row), red channel (second row) and merged + brightfield image (bottom row). While the fluorescein control only penetrates the upper infundibulum of the hair follicle (green channel), penetration is extended to deeper areas of the infundibulum after the addition of 2% PSNPs (red channel). After the addition of 4% PSNPs, the penetration of fluorescein is further extended into the isthmus area, while there is no further significant increase after the addition of 8% PSNPs indicating a saturation of follicular penetration depth between 4% and 8%

Interestingly, a higher penetration depth can be achieved if the concentration of PSNPs is increased. For example, significantly higher penetration values were achieved with a PSNP content of 4 or 8% than with a pure drug solution or a particle content of only 2%. From the results it can be concluded that the follicular penetration depth of fluorescein was extended from the infundibular area (control) to the isthmus area mediated through the added PSNPS (4% and 8% concentrations) meaning that the upper third of the hair follicle can be targeted (Fig. 3). This area represents the section from the sebaceous gland excretory ducts to the exit of the hair follicle and is therefore rich in sebum. This results in a wide range of possible applications like acne treatment, skin antisepsis or even the transfollicular delivery of several dermal therapeutics and agents against alopecia. Since in this fraction the most microbes can be found, one of the most interesting fields of application of this technology is the decontamination of hair follicles, as already published in other papers [7–11]. The aim is to eliminate the intrafollicular microbiome through the particle-mediated transport of antiseptics into deeper areas of the hair follicles and thus prevent recolonization of the skin surface, e.g. during prolonged surgical procedures.

In previous studies, however, the particles were loaded with an antiseptic. Therefore, the manufacturing process involves some hurdles, and due to the complexity of drug delivery systems resulting from the inclusion of trigger-responsive materials and possible material incompatibilities, the systems often end up being unstable.

However, the method presented here circumvents these problems, which is why it would be particularly interesting for preoperative skin decolonization. In this scenario the NPs could be added to an antiseptic solution preoperatively to produce a ready-to-use suspension.

**Fig. 4:**
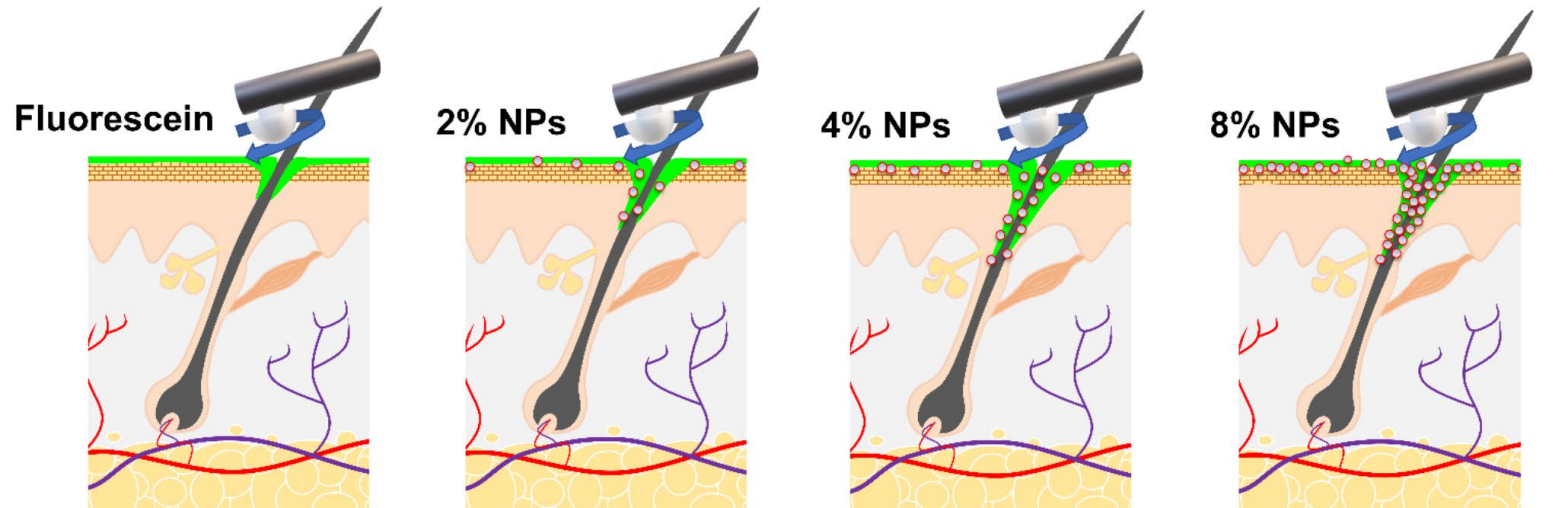
Schematic representation of particle-assisted penetration of active ingredient solutions. The addition of 2% NPs leads to a slight increase in the penetration depth due to an adhesive effect in which the NPs, which are present in the formulation independently of the solution, draw the solution with the active ingredient dissolved in it into the deeper sections of the hair follicles through an adhesive effect. This effect becomes significant when the NP content is doubled to 4%, but does not show any significant differences compared to an 8% content. This saturation effect could be caused by crowding of the NPs in the infundibulum of the hair follicle, which prevents effective transport of further NPs into deeper sections of the hair follicle

With this work, we were able to demonstrate that a saturation effect with regard to drug transport in the hair follicle occurs between 4% and 8% PSNP content (Fig. 4). With respect to the future production of such systems, this statement is of great importance. While doubling the NP concentration from 2 to 4% ensures that more particles are effectively transported to deeper areas of the hair follicle, thus drawing the outer phase deeper into the hair follicle, a limitation of the follicular penetration depth in the context of the mentioned saturation could be caused by a crowding effect of the particles, which hinders effective free transport into the depth of the hair follicle and thus also of the active ingredient solution.

An increase in the intrafollicular concentration represented by the fluorescence intensity of the model compound fluorescein transported into the hair follicle could not be demonstrated in this work. This is probably due to the fact that an increased penetration depth leads to a gradient of fluorescence intensity in the lower fractions of fluorescence signal. An increased penetration depth may therefore also result in sections with lower fluorescence intensity, which reduces the overall fluorescence intensity of the area of interest. We therefore propose an adapted method for a fully quantitative assessment. Here, for example, a biopsy of the hair follicle by plucking the hair and dissolving the follicle contents could be used for quantification. This method will be subject of our future work. In addition, the application of an in vivo study to demonstrate the particle-mediated enhancement of antiseptic effects based on reduced recolonization of the skin surface by pathogens originating from the deeper parts of the hair follicles is a necessary translational approach for a future clinical application of this drug delivery system, as ex vivo porcine skin fails as a model for skin recolonization due to the lack of sebum flow.

## Conclusions

In this publication it could be shown that 4% NPs significantly enhance the transport efficiency of the model drug fluorescein, solved in the outer phase of the formulation, in the framework of particle-mediated transport. Doubling the concentration to 8% did not result in a significant increase of follicular penetration depth, which may be based on an intrainfundibular crowding effect. The increase of follicular penetration depth evolved more efficiently with a 4 Hz massage application as compared to 100 Hz oscillating massage. These results form the basis for optimal formula proportions as well as optimal application parameters for a future application in clinical studies for e.g. skin antisepsis purposes.

## Data Availability

The datasets generated during and/or analysed during the current study are available from the corresponding author on reasonable request.
